# Screening and Characterization of Sialic Acid-Binding Variable Lymphocyte Receptors from Hagfish

**DOI:** 10.3390/biotech13040046

**Published:** 2024-11-12

**Authors:** Mark Rickard N. Angelia, Abigail Joy D. Rodelas-Angelia, Cheolung Yang, Sojeong Park, Seung pyo Jeong, Hyeok Jang, Dennis Berbulla Bela-ong, Hobin Jang, Kim D. Thompson, Taesung Jung

**Affiliations:** 1 Laboratory of Aquatic Animal Diseases, Institute of Animal Medicine, College of Veterinary Medicine, Gyeongsang National University, 501-201, 501, Jinju-Daero, Jinju-si 52828, Gyeongsangnam-do, Republic of Korea; mrna@gnu.ac.kr (M.R.N.A.); ajrangelia@gnu.ac.kr (A.J.D.R.-A.); j6acjfdnd@naver.com (C.Y.); pkrso82@naver.com (S.P.); jspye1@gmail.com (S.p.J.); jangm1379@gmail.com (H.J.); dbelaong@yahoo.com (D.B.B.-o.); 2 Institute of Chemistry, College of Arts and Sciences, University of the Philippines Los Baños, College, Laguna 4031, Philippines; 3 Center for Study of Emerging and Re-Emerging Viruses, Korea Virus Research Institute, Institute for Basic Science, Daejeon 34126, Republic of Korea; hobin1976@gmail.com; 4 Moredun Research Institute, Pentlands Science Park, Bush Loan, Midlothian EH26 0PZ, UK; kim.thompson@moredun.ac.uk

**Keywords:** variable lymphocyte receptors, hagfish VLRB, sialic acid binding, glycan recognition, ccombodies

## Abstract

Sialic acid is a diverse group of monosaccharides often found on the termini of *N*- and *O*-linked glycans as well as being components of glycoconjugates. Hypersialylation has been associated with the progression of chronic inflammation-mediated diseases such as cardiovascular disease and cancer. Given its role in infection and disease-related processes, sialic acid is a promising target for therapeutic approaches that utilize carbohydrate-binding molecules. In this study, we screened for sialic acid-recognizing variable lymphocyte receptors (VLRBs) or ccombodies from inshore hagfish (*Eptatretus burgeri*) using a synthetic Neu5Ac-glycoconjugate as an antigen in immunoassay. Resulting ccombodies, 2D8, 5G11, 4A1, and 5F8 were further characterized in terms of their binding activity and specificity. A competitive ELISA using free haptens showed strong inhibition using either *N*-acetylneuraminic acid (Neu5Ac) and *N*-glycolylneuraminic acid (Neu5Gc). The half-maximal inhibitory concentrations (IC_50_) for Neu5Ac ranged from 7.02 to 17.06 mM, with candidates 4A1 and 5G11 requiring the least and highest amounts, respectively. IC_50_ values for Neu5Gc ranged from 8.12 to 13.91 mM, for 4A1 and 5G11, respectively. Candidate ccombodies also detected naturally occurring sialic acid from known sialoglycoproteins using a dot blot assay. Neu5Gc-5G11 and Neu5Ac-2D8 yielded the strongest and weakest docking interactions with affinity values of −5.9 kcal/mol and −4.9 kcal/mol, respectively. Hydrogen bonding and hydrophobic interactions were predicted to be the predominant noncovalent forces observed between the ccombodies and sialic acid. This study demonstrates that glycan-binding VLRBs from hagfish hold promise in augmenting the glycobiologists’ toolkit in investigating the roles of glycans in human and animal health and disease.

## 1. Introduction

Jawless vertebrates (agnathans), such as lamprey and hagfish, lack conventional immunoglobulin-based antibodies normally found in jawed vertebrates. Instead, they rely on an ancient kind of protein receptor, known as variable lymphocyte receptors (VLRs), for their humoral- and cell-mediated adaptive immune responses. In general, VLRs are characterized by a solenoid 3D structure built from several leucine-rich repeat (LRR) modules [1].

Five VLR genes have so far been discovered, namely (a) VLRA, (b) VLRB, (c) VLRC, (d) VLRD, and (e) VLRE. Both VLRA and VLRC are assembled in the thymoid, which are structures akin to the thymus, and expressed solely as transmembrane proteins having roles analogous to the T-cell receptors of jawed vertebrates. VLRB, on the other hand, is considered the B-cell receptor counterpart of jawed vertebrates and is expressed both as a membrane-bound or secreted protein. Similarly, VLRD and VLRE demonstrate T-cell-like functions and are abundantly expressed by lymphocytes (triple-negative VLRA^−^/VLRB^−^/VLRC^−^) devoid of the other VLR types and to a lesser extent in VLRA^+^ and VLRC^+^ cells [2,3,4].

Of the different kinds of VLRs, VLRB has the most promising range of applications due to its soluble and immunoglobulin-like nature. As a single polypeptide product, gene manipulation and engineering of VLRBs with improved affinity and specificity entail fewer complications as opposed to the traditional monoclonal antibody-based technologies [5]. While there are subtle structural differences in secreted forms of VLRBs from lamprey and hagfish [6], both species’ VLRBs have been harnessed for therapeutic development [7,8,9,10] as well as pathogen recognition and neutralization in agriculture [11,12] and aquaculture [13,14,15,16].

On the other hand, glycans are ubiquitous in biological systems. They can exist as simple monosaccharides, or linked together to form polysaccharides, and may even be conjugated to other biomolecules forming a plethora of glycoconjugates, such as glycolipids, glycoproteins, proteoglycans, and glycosaminoglycans. Their multifunctional roles include acting as substrates for energy production, recognition molecules for intercellular communications, and structural components [17,18]. Recently, however, aberrant glycosylation patterns and alterations in glycan metabolism were revealed to be hallmarks of emerging diseases related to tumor progression, inflammatory response, bacterial and viral infection, etc. [19,20,21]. In cancer, the overexpression of enzymes, namely sialyltransferases and fucosyltransferases, has led to enhanced synthesis of tumor-associated carbohydrate antigens (TACAs), further leading to metastasis and increased mortality [22]. In cancer, this hypersialylation event favors angiogenesis, immune response evasion, therapy and apoptosis resistance [23]. Furthermore, sialic acid also acts as a marker for cardiovascular and related diseases [24]. Therefore, it is imperative to develop effective strategies for the reliable detection of sialic acid in glycoconjugates due to its vital role in both health and disease.

In this study, we screened an existing inshore hagfish (*Eptatretus burgeri*) VLRB (henceforth also referred to as “Ccombody”) library for the presence of sialic acid-binding receptors. This is the first report of glycan-binding ccombodies identified and isolated from hagfish.

## 2. Methodology

### 2.1. Synthesis of Sialoglycoconjugate

The synthesis of sialoglycoconjugate was adopted from the methods of Gray [25] and Abuknesha et al. [26], with modifications. *N*-acetylneuraminic acid (Neu5Ac) was conjugated to bovine serum albumin (BSA) using cyanuric chloride (CC) as a chemical linker. To accomplish this, 1 mL of carbonate–bicarbonate buffer (pH 9.0) was initially added with 100 μL of a solution containing 2 mg Neu5Ac, followed by brief vortexing. To this solution, 1 mg of CC was then added, and the reaction was allowed to proceed for 2 h at 37 °C inside a rotary shaker. After activation of Neu5Ac with CC, 100 μL of a 1 mg BSA solution was added, and the mixture was kept overnight at 4 °C. Unreacted sugar and linker were removed by subjecting the mixture to ultrafiltration for 15 min using Amicon Ultra 0.5 mL centrifugal filters, with a 10,000 MW cutoff (Merck Millipore, Darmstadt, Germany). The ultrafiltration column was washed twice with ultrapure distilled water, then the filtrate was resuspended to a final volume of 500 μL using ultrapure distilled water. Glycoconjugate formation was verified through SDS-PAGE and periodic acid Schiff (PAS) staining (Thermo Fisher Scientific, Waltham, MA, USA) using 10% polyacrylamide gel. The extent of conjugation was quantified using the total carbohydrate assay kit (Abcam, Cambridge, UK) according to the manufacturer’s specifications.

### 2.2. ELISA Screening of VLRB Library

ELISA was carried out according to the method of Lazarte et al. [15], with some modifications. Screening for sialic acid-binding ccombodies was carried out by coating 100 μL of a 300 ng/well BSA-CC-Neu5Ac glycoconjugate onto high-binding 96-well ELISA plates (Corning, Glendale, AZ, USA), along with BSA only (without CC) as a negative control. Plates were left standing overnight at 4 °C, after which they were washed three times with 1X TBST (10 mM Tris-HCl, 150 mM NaCl, and 0.5% Tween) to remove unbound antigen. Plates were blocked for 30 min with blocking buffer comprising 5% skimmed milk in 1× TBST at room temperature, then washed once with 1X TBST. Ccombody supernatants from HEK293F cells were then added onto the plates, incubated for 1 h 30 min, followed by washing three times with 1X TBST. Antigen bound ccombodies were detected with mouse anti-VLRB antibody (11G5) in blocking buffer by incubation for 1 h, followed by washing three times to remove unbound 11G5. Finally, bound 11G5 antibodies were detected by incubating for 1 h with HRP-conjugated goat anti-mouse IgG (1:5000 *v*/*v* in blocking buffer), then washed four times. Binding was quantified by the addition of 100 μL developing buffer composed of 42 mM 3,3′,5,5′-tetramethylbenzidine and 1% H_2_O_2_, then color development for 30 min at room temperature. The reaction was stopped by adding 50 μL of 2 M H_2_SO_4_. The absorbance was then read at 450 nm using an xMark (BIO-RAD, Hercules, CA USA) microplate reader.

### 2.3. Cloning and Expression of VLRBs in HEK293F Cells

The ccombodies used in this study contained a C4bp multimerization domain designed to increase VLRB stability and avidity [11]. The initial step for isolation of the HEK293F clones expressing the desired heptamerized sialic-acid binding VLRBs entailed pelleting the cells by centrifugation at 5000 rpm for 3 min then subsequent lysis using 40 μL of 0.02 N NaOH. A 1 μL of the lysate was used as a template for PCR using primers LRRNT *Sfi I* and Stalk *Sfi I* with the following parameters: initial denaturation at 95 °C for 3 min, denaturation at 95 °C for 20 s, annealing at 60 °C for 10 s, extension at 72 °C for 40 s, and final extension at 72 °C for 3 min. PCR amplicons were later purified using a DNA purification kit (iNtRON Biotechnology, Seongnam-si, Republic of Korea), and then excision sites were introduced by incubation with *Sfi I* in CutSmart buffer (New England Biolabs, Ipswich, MA, USA) for 2 h at 50 °C. Plasmid ∆514/kepta vector (designed by our lab) was similarly digested following the previous conditions. Ligation of amplicons and vectors was carried out at room temperature for 30 min using a ligation reaction premix (Bioneer, Daejeon, Republic of Korea). Next, HEK293F cells in 24-well plate were transfected with the assembled plasmids using Lipofectamine 2000 (Thermo Fisher Scientific, Waltham, MA, USA). After 4 h, the transfection medium was replaced with expression medium (Thermo Fisher Scientific, Waltham, MA, USA). Culture supernatants were then screened for the presence of antigen-specific ccombodies after 72 h using the ELISA previously described.

### 2.4. Determination of Relative Dissociation Constant

The relative dissociation constant (K_D_) was determined through the ELISA presented earlier but with some modifications. Antigen coating was performed using a fixed amount at 500 ng/well added with varying amounts of ccombody-containing culture supernatants, serially diluted two-fold (1 to 1024×) using 5% skimmed milk in 1X TBST. The background corrected OD_450_ values were plotted against the ccombody dilution, and the relative K_D_ and Hill coefficient were computed for each candidate by fitting the ccombody titration data into the Hill equation. Values were reported as the mean of two measurements.

### 2.5. Competitive Inhibition Assays and IC_50_ Determination

Competitive inhibition was determined through ELISA as presented earlier, with some modifications. Optimal subsaturating dilutions of ccombody-culture supernatants were used based on the preliminary antibody titration data. Competitive inhibitors, two-fold serially diluted (50 to 0.39 mM), were mixed with a constant amount of ccombody supernatant diluted in 5% skimmed milk in 1X TBST, then incubated for 1 h at room temperature. Next, 100 μL of the mixture was subsequently transferred into another 96-well ELISA plate containing a constant amount of antigen (500 ng/well) which was pre-blocked with 5% skimmed milk in 1X TBST. Antigen-bound ccombodies were detected by 11G5 and HRP-conjugated goat anti-mouse IgG. The normalized and background-corrected OD_450_ values were plotted against the ligand concentration, and the half-maximal inhibitory concentration (IC_50_) was computed by curve fitting using the Four Parameter Logistic (4PL) Curve Calculator [27]. Values are expressed as the mean of three determinations.

### 2.6. Western and Dot Blot Assays Using Ccombody Candidates

The specificity of ccombody candidates was validated using western and dot blot assays against BSA-CC-Neu5Ac and sialoglycoproteins, respectively. For Western blot assay, the BSA-CC-Neu5Ac was first resolved in 10% SDS-PAGE under reducing conditions and proteins then transferred onto a nitrocellulose membrane using a Trans-Blot Turbo Transfer System (BIO-RAD, Hercules, CA, USA). Blocking of the membrane was performed using 5% skimmed milk in phosphate-buffered saline (PBS) with 0.1% Tween 20, incubating for 1 h with ccombody candidates. Bound ccombodies were detected by successive incubations with mouse anti-VLRB antibody (11G5) and HRP-conjugated goat anti-mouse IgG, both used in blocking buffer. Blots were developed by immersing the membrane in SuperSignal West Pico PLUS Chemiluminescent substrate (Thermo Fisher Scientific, Waltham, MA, USA). Dot blot assays were performed in a similar manner, except 1 μL of 10 mg sialoglycoproteins was directly blotted onto the nitrocellulose membrane. Dot quantification by densitometric analysis was carried out using Fiji (ImageJ2 v.1.54f) software [28]. Background subtraction and dot selection using the rectangular selection tool were performed for each image. Dot densities were then expressed as percentage of area intensity.

### 2.7. Molecular Docking of Ccombody Candidates Using Neu5Ac and Neu5Gc as Ligands

The docking studies were carried out using monomerized forms of the ccombody candidates against the ligand. The structures of ccombodies 2D8, 4A1, 5G11, and 6D2 were generated using AlphaFold2 [29]. Top-ranked structures, those with the highest pLDDT (per-residue log likelihood derived from deep learning true), and pTM (predicted template modeling) scores, were selected for docking studies. Putative ligand binding sites were verified using the PrankWeb 3 P2Rank server (Figure S1) [30]. On the other hand, ligand structures were downloaded from Pubchem [31]. Molecular docking between the ccombody receptor and glycan ligand was accomplished via AutoDock Vina v.1.2 through the SeamDock server [32]. Docking results and molecular interactions were visualized via ChimeraX v.1.8 [33] and Ligplot+ v.2.2 [34], respectively.

## 3. Results

### 3.1. Verification of Conjugation

**Figure 1:**
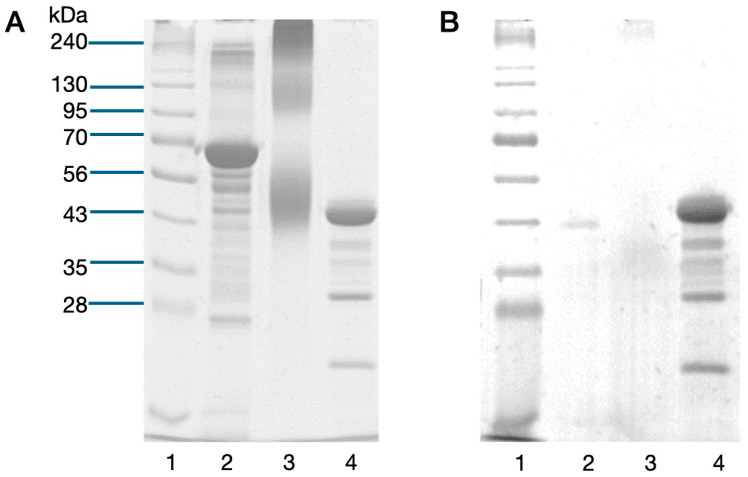
(**A**) SDS-PAGE profile of BSA-CC-Neu5Ac conjugate, (**B**) PAS stain of BSA-CC-Neu5Ac. Lane 1—MW marker, 2—BSA, 3—BSA-CC-Neu5Ac, 4—HRP (PAS staining positive control).

Sialic acid, in the form of *N*-acetylneuraminic acid, was conjugated to BSA using cyanuric chloride as the chemical linker. The average total carbohydrate content of the conjugate was 0.011 μg carbohydrate/μg protein. The dispersed bands in the SDS-PAGE profile, shown in Figure 1, imply that the conjugation occurred to various degrees for different BSA molecules. Glycoprotein staining shows smeared bands with the BSA-CC-Neu5Ac conjugate at a size of approximately >240 kDa. However, lower molecular weight conjugates may be present, but their amounts were below the detection limit of the staining method.

### 3.2. Antibody Titration and Dissociation Constant

**Figure 2:**
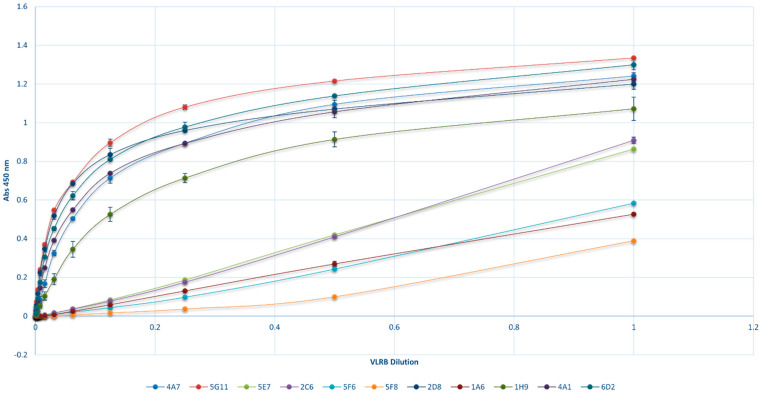
Antibody titration of various ccombodies against BSA-CC-Neu5Ac. Values are the average of two determinations, with the standard deviation shown as error bars.

BSA-CC-Neu5Ac was then used as the antigen for screening sialic acid-binding ccombodies from an existing hagfish VLRB library (developed in our lab). We identified eleven candidates that showed promising specificities and affinities during the initial assays. As expected, antibody titration revealed a proportional relationship between binding response and ccombody concentration from the cell culture supernatants (Figure 2). Consequently, this allowed for a rapid comparison of ccombody affinities, without the need for further purification of each candidate, simply by expressing the dissociation constant (K_D_) as relative values in terms of the dilution factor (Figure 2 and Table 1). High affinity ccombody candidates were characterized by hyperbolic plots, with relative K_D_ values below 1. Conversely, candidates with low affinities yielded more linear plots, with K_D_ values above 1. Ccombodies 2D8 and 5F8 had the lowest and highest relative K_D_ values of 0.055 and 14.103 corresponding to the strongest and weakest affinities, respectively. Except for candidate 5F8, the rest of the ccombodies have a Hill coefficient close to 1, signifying a non-cooperative binding behavior between each receptor-ligand pair.

### 3.3. Competitive Inhibition and Western Blot Assays

**Figure 3:**
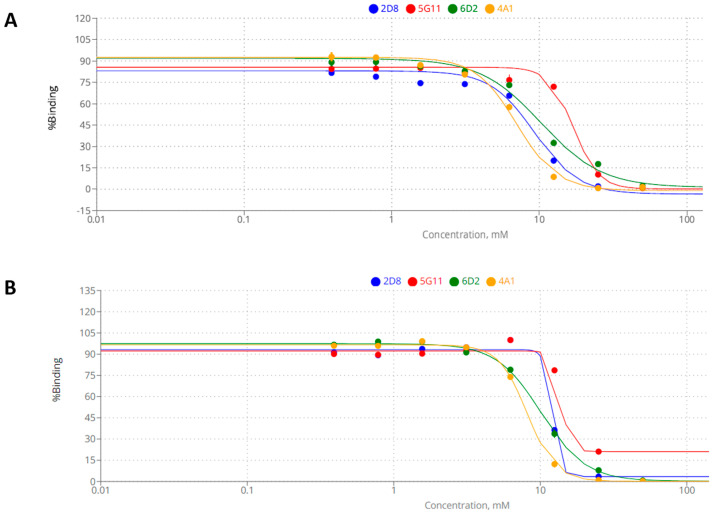
Competitive ELISA of ccombody candidates against (**A**) Neu5Ac and (**B**) Neu5Gc.

For the succeeding experiments, only the top four candidates with highest affinities, namely 2D8, 4A1, 5G11, and 6D2, were subjected to further characterizations and competitive ELISA (Figure 3). Free haptens were used to assess the specificity of each ccombody, and concomitantly eliminate the probability of the carrier protein and chemical spacer being part of the recognition motif. Monosaccharides, especially those commonly incorporated in *N*- and *O*-glycans in glycoproteins, were chosen as soluble ligands acting as competitive inhibitors.

The two most common forms of sialic acid, Neu5Ac and Neu5Gc (*N*-glycolylneuraminic acid), yielded strong inhibitory effects on all four candidates (Figure 3), with their half-maximal inhibitory concentrations (IC_50_) shown in Table 2.

The Neu5Ac concentrations necessary to achieve half-maximal inhibition ranged from 7.02 to 17.06 mM, with candidates 4A1 and 5G11 requiring the least and highest amounts, respectively. On the other hand, for Neu5Gc, the values ranged from 8.12 to 13.91 mM, with candidates 4A1 and 5G11 requiring the least and highest amounts, respectively. No significant inhibition was observed for the other monosaccharides tested, namely mannose, glucose, galactose, *N*-acetylgalactosamine, and *N*-acetylglucosamine (Figure S2).

**Figure 4:**
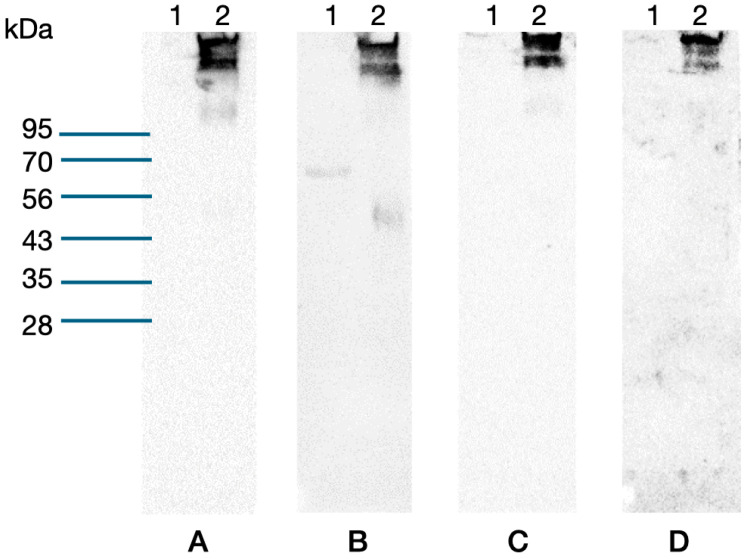
Western blot assays of BSA-CC-Neu5Ac using candidates (**A**) 2D8, (**B**) 5G11, (**C**) 6D2, and (**D**) 4A1. Lane 1—BSA, 2—BSA-CC-Neu5Ac.

Furthermore, Western blot assays were employed to validate the results from the hapten inhibition assays (Figure 4). Although the four ccombodies showed the desired selectivity to the sialoglycoconjugate, 5G11 appears to weakly recognize BSA, implying that a portion of the carrier protein may form part of the epitope.

### 3.4. Dot Blot Assay Using Sialoglycoproteins

**Figure 5:**
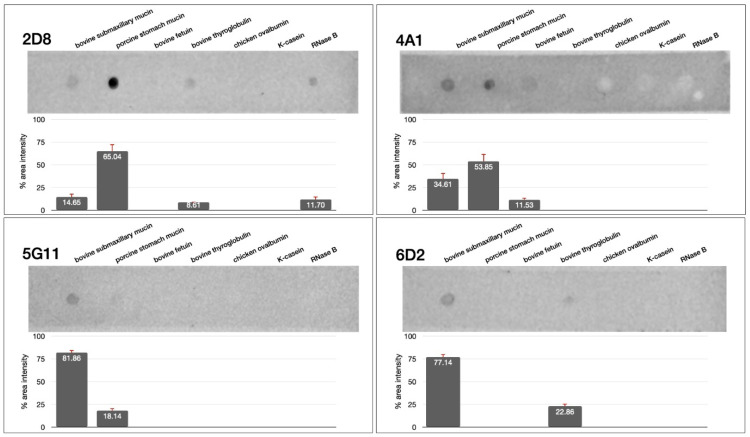
Representative dot blot assay and percent area intensity of ccombody candidates 2D8, 4A1, 5G11, and 6D2 against known sialoglycoproteins. Percent area intensity for each dot was obtained using densitometric analysis.

One of the challenges of using artificial linkers like CC is the possibility of false-negatives, attributed to the linker-dependent alteration of the glycan’s topology and recognizability. Artificial linkers tend to display glycans in a manner different from their native conformations [35]. Hence, we examined if our ccombody candidates could recognize naturally occurring sialic acid using dot blot assays against various sialoglycoproteins such as bovine submaxillary mucin [36], bovine fetuin [37], bovine thyroglobulin [38], chicken ovalbumin [39], porcine stomach mucin [40], K-casein [41], and RNase B [42]. The ccombody candidates recognized and stained the sialoglycoproteins to varying degrees. For instance, 2D8 and 4A1 predominantly recognized porcine stomach mucin, with over 50% of the area intensity attributed to the dot density. On the other hand, bovine submaxillary mucin was strongly recognized by 5G11 and 6D2, yielding over 75% of the area intensity (Figure 5). Bovine thyroglobulin was weakly recognized by both 2D8 and 6D2, while bovine fetuin and RNase B were recognized by 4A1 and 2D8, respectively. The results suggest that recognition of sialic acid may be influenced by other factors such as residue density and clustering, display conformation, moiety alteration, steric effects by underlying glycan residues, and the nature of detection platform [35,43]. Nonetheless, this demonstrates the detection abilities of the ccombodies against naturally occurring sialic acid in glycoproteins.

### 3.5. Binding Interactions Between Sialic Acid and Ccombodies

Despite the lack of crystallographic data, in silico molecular docking studies permitted us to probe the significant stabilizing interactions between the ccombodies and sialic acid. Unbound sialic acid was used during docking to reflect the observed binding behavior of the monosaccharides (Neu5Ac and Neu5Gc) during competitive ELISA assay. Sialic acid in solution inherently undergoes mutarotation to yield the α and β anomers [44]. Hence, both the α and β anomeric forms were allowed to bind, depending on the preferred docking pose of the ligand. This also simplifies the docking protocol without the need to specify the torsion angles associated with glycosidic linkages in oligosaccharides.

**Figure 6:**
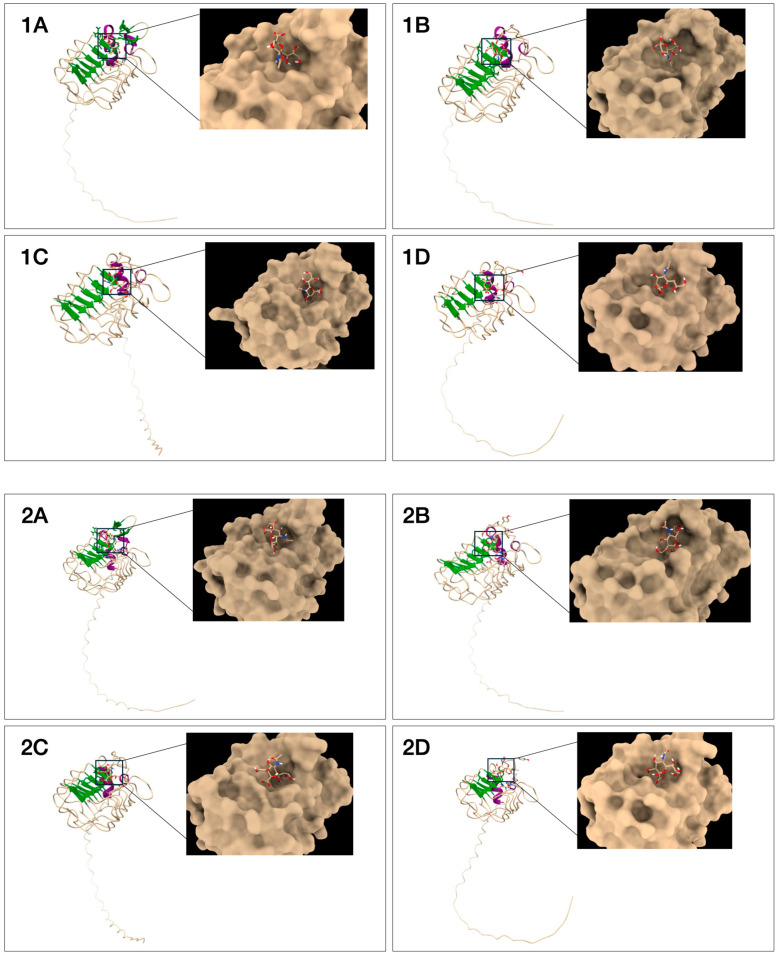
Molecular docking of ccombody candidates (**A**) 2D8, (**B**) 5G11, (**C**) 6D2, and (**D**) 4A1 with (**1**) Neu5Ac and (**2**) Neu5Gc using AutoDock Vina. Protein structures are represented using a ribbon model (left) and a space-filling model (right) while the glycan ligand is represented by a stick model.

The ligand poses presented in Figure 6 were selected based on their binding affinities (Table 3), with more negative values indicating more favorable ligand conformations and stronger receptor–ligand interactions. Neu5Gc bound to ccombody 5G11 showed the highest docking affinity, with a score of −5.9 kcal/mole, while Neu5Ac bound to ccombody 2D8 yielded the weakest affinity with −4.9 kcal/mole.

**Figure 7:**
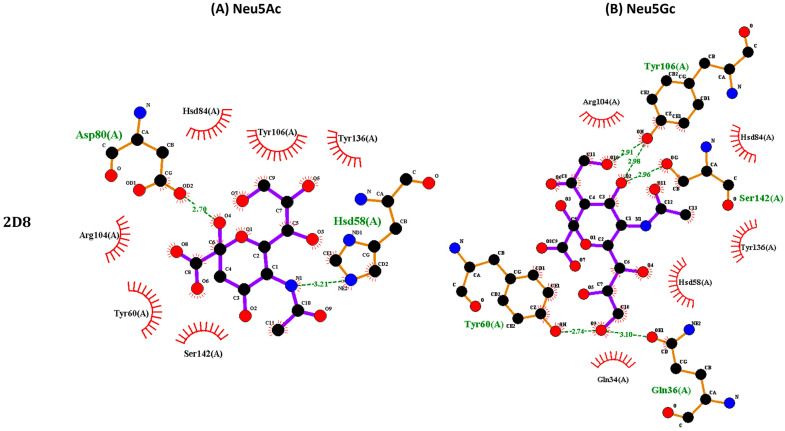

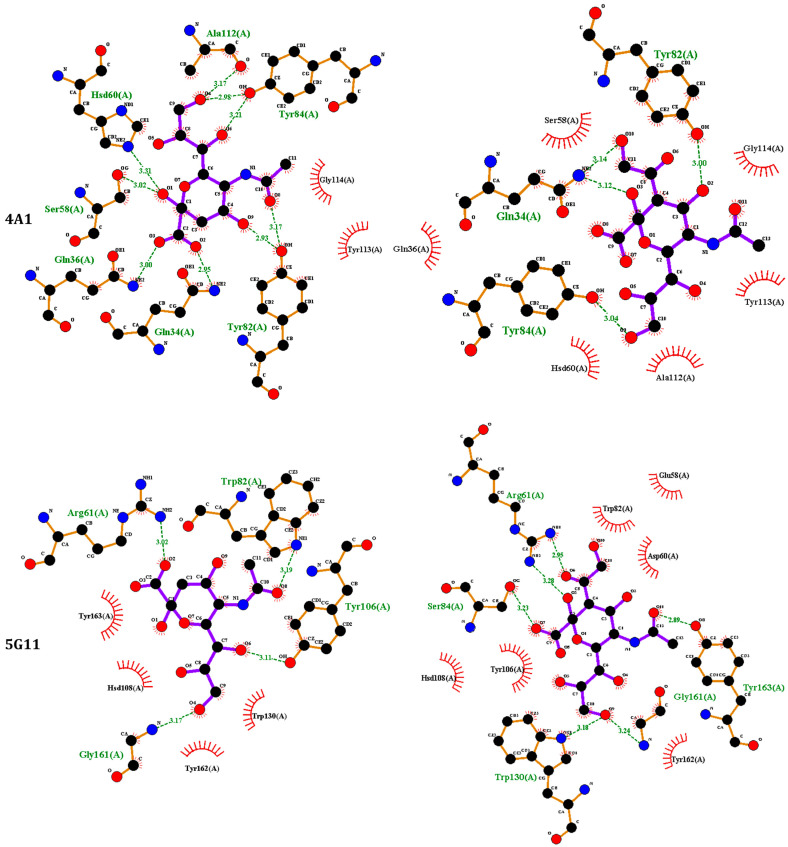

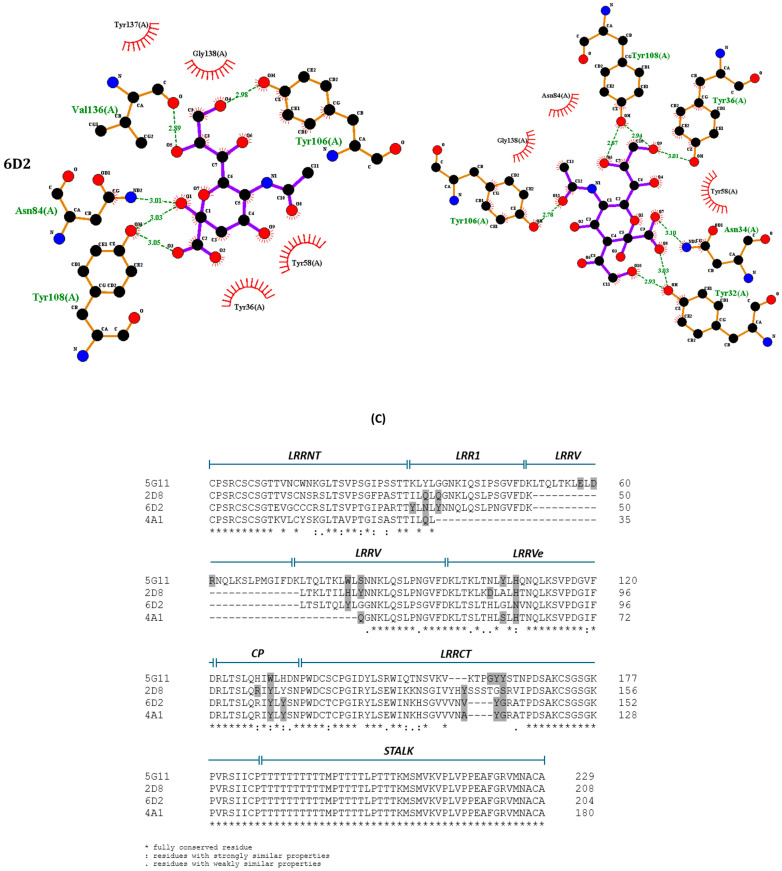
Noncovalent interactions between ccombodies and sialic acids (**A**) Neu5Ac and (**B**) Neu5Gc. Amino acid residues involved in hydrogen bonding (green dashed lines) are colored green. Red spoked arcs are residues that exert hydrophobic interactions. (**C**) Multiple sequence alignment of sialic acid-binding ccombodies. Residues in gray are positioned in the LRR hypervariable region and predicted to interact with the ligand.

All four ccombody candidates were seen to possess a deep pocket on the putative binding site located on the concave face, allowing Neu5Ac and Neu5Gc to fit snugly (Figure 6). In addition, the ccombodies exhibited features typical of glycan-binding VLRBs discovered thus far, namely (1) the proximity of the receptor pocket to the LRRCT domain, preference for aromatic amino acids, Asn, and Asp in the LRR hypervariable positions, and exploitation of multiple noncovalent interactions for ligand binding (Figure 6 and Figure 7). Predominant binding interactions that were noted include hydrogen bonding and hydrophobic interactions. As expected for glycans, the multiple hydroxyl groups typically present in the structure favor the formation of hydrogen bonds with polar and charged amino acids. In contrast, hydrophobic interactions emanate from aromatic amino acids that confer CH-π or stacking interactions with the glycan ring, aside from decorating the binding pocket with hydrophobic patches (Figure 7 and Table 3) [45]. Multiple sequence alignment shows that ccombody 5G11 has the highest number of LRR modules, and consequently, the greatest number of interacting amino acids, amounting to 11 residues, while ccombody 4A1 has the smallest number of LRR modules and only nine interacting amino residues. Surprisingly, no predicted interactions were found for residues belonging to the LRRNT module for all ccombodies, which contrasts those exhibited by lamprey VLRBs against their glycan ligands [7,43].

## 4. Discussion

The sialic acid monosaccharide family is an expansive group comprising more than 50 members, with certain members often decorating the termini of glycans in glycoconjugates such as glycoproteins, glycolipids, and proteoglycans. Their position as outermost glycans is implicated in numerous physiological processes such as cell recognition and signaling, disease progression, and immune modulation, making them ideal targets for detection and analysis using glycan-binding proteins [22]. Thus, this study sought to identify and characterize glycan-binding ccombodies, particularly sialic acid-recognizing receptors, from an existing hagfish VLRB library. To circumvent the difficulty of immobilizing monosaccharides for ccombody screening and optimize antigen presentation, we constructed a sialoglycoconjugate, with BSA as a carrier protein and CC as a chemical linker, to serve as antigen in ELISA screening. We believe that CC is an ideal linker as it provides the necessary multivalency and is well suited for the multimeric nature of our developed VLRB, supporting at most two glycan molecules per linker, with ample directionality and free rotation for each linked glycan [46,47,48]. Typically, small glycans are poorly immunogenic; hence, conjugation to various platforms to create synthetic glycoconjugates has become a promising strategy which is already being employed extensively for identifying GBPs like monoclonal antibodies, lectins, and cellular and artificial receptors [49,50,51]. For instance, Hong et al. [52] screened for lamprey VLRB glycan-binders by utilizing a high throughput technique involving glycan-polyacrylamide (PAA) conjugates and yeast surface display (YSD) libraries of VLRs, in combination with MACS and FACS enrichment. On the other hand, Ward et al. [53] identified glycan-binding proteins from a YSD library comprising reduced charge variants of a DNA-binding protein scaffold Sso7d, utilizing glycans coupled to either magnetic beads or PAA.

To date, all known glycan-binding VLRs have been isolated from lamprey, with glycotopes varying in size and complexity, ranging from simple monosaccharides to more complex oligo- and polysaccharides [52,54]. The modular nature of the LRR domains enables the VLRs to bind numerous glycan determinants simply by varying the quantities of LRR modules. While smaller VLRs, containing fewer LRR modules, tend to bind smaller glycotopes, larger ones with more modules can accommodate longer and more complex determinants. Furthermore, glycan-binding VLRs take advantage of CH-π or hydrophobic stacking forces from their LRRCT loops to lock the glycotopes firmly in place, enclosing the glycan between the concave-binding domain and the LRRCT module. In lamprey VLRs, this hydrophobic stacking interaction is conferred by a conserved tryptophan, an aromatic amino acid, that protrudes above the glycan-binding pocket [54]. Interestingly, the structural features described are similarly mirrored by our sialic acid-binding ccombodies. Molecular docking and in silico analyses revealed that the binding pocket is constructed from amino acids positioned in the hypervariable sites that are in proximity to the LRRCT loop. This observation is further supported by the lack of predicted amino acid interactions from the more distant LRRNT module. However, unlike the lamprey receptor, hagfish ccombodies employ mainly a tyrosine residue in the LRRCT domain for the stacking force in place of the tryptophan moiety. Moreover, consistent with our previous studies, the invariant stalk region of hagfish ccombodies appears to be devoid of direct interaction with antigens, a notion that may further extend to glycotopes [11].

While our ccombodies demonstrated sufficient selectivity and specificity for sialic acid (Neu5Ac and Neu5Gc) as assessed by hapten inhibition and Western blot assays, it is worth noting that the candidates were unable to discriminate between the two closely related structures. Neu5Gc, a hydroxylated version of Neu5Ac, is found naturally in mammals except for humans due to the inactivation of the CMP-*N*-acetylneuraminic acid hydroxylase (CMAH) enzymes responsible for its biosynthesis [55]. Although humans can still acquire Neu5Gc from their diet, its accumulation has been associated with the development of various chronic inflammation-mediated diseases and poses a hurdle in xenotransplantation [56,57]. This cross-reactivity between very closely related glycan structures against the ccombodies is a behavior often observed in a wide range of natural and artificial glycan-binding receptors and remains a challenge that is yet to be addressed through the systematic design of glycan derivatives and protein engineering [58].

The significance of glycobiology is fully recognized only when a comprehensive set of tools is available for detecting and thoroughly analyzing the complete range of natural glycan diversity. Molecules that can detect changes in normal glycan presentation or composition would, therefore, be functionally relevant especially since changes in glycan patterns are implicated in various infectious diseases [59]. Aberrant sialylation patterns, for example, are associated with cancer and autoimmune disorders since pathogens rely on sugars for host invasion or immune evasion [22,60]. Limitations often encountered by those engaged in this field are the broad specificities exhibited by glycan-binding reagents, such as monoclonal antibodies or lectins, as well as the absence or poor binding affinities to selected glycan-epitopes [7,61,62]. Additionally, the production of antibodies using hybridoma technology is limited by the weak immunogenicity of mammalian glycans from humans and mice, attributable to the similarity between the glycans from these sources. These inherent challenges posed by working with ultra-diverse structures opened an exciting area where alternate platforms for glycan analysis can be explored. McKitrick and colleagues [54] developed Smart Anti-Glycan Reagents (SAGRs) using lamprey VLRBs and have successfully taken advantage of evolutionarily distant but functionally similar receptors to augment the workflow. Likewise, this study on ccombodies provides a window of opportunity for the discovery of more glycan-recognizing VLRBs from hagfish. We have demonstrated in this study the strong potential for hagfish VLRBs as a source of glycan-binding proteins for both synthetic and naturally occurring glycoconjugates.

## Figures and Tables

**Table 1:** Affinities of the ccombodies expressed as relative K_D_ values.

Candidate	Relative K_D_	Hill Coefficient
2D8	0.055	0.806
5G11	0.074	0.750
6D2	0.099	0.777
4A1	0.120	0.777
4A7	0.122	0.941
1H9	0.190	0.950
1A6	2.204	1.207
5E7	2.890	1.234
2C6	7.840	1.219
5F6	14.103	1.290
5F8	22.014	1.863

**Table 2:** Half-maximal inhibitory concentrations of Neu5Ac and Neu5Gc from competitive ELISA.

Candidate	IC_50_, mM	
	Neu5Ac	Neu5Gc
4A1	7.02	8.12
2D8	9.26	12.07
6D2	10.26	10.10
5G11	17.06	13.91

**Table 3:** The affinity between ccombody candidates and sialic acid. Amino acid residues involved in significant noncovalent interactions are listed with their positions indicated.

Ccombody	Sialic Acid	Affinity (kcal/mol)	Hydrogen Bonding	Hydrophobic Interactions
2D8	Neu5Ac	−4.9	Asp-80, Hsd-58	Tyr-60, Hsd-84, Arg-104, Tyr-106, Tyr-136, Ser-142
	Neu5Gc	−5.0	Gln-36, Tyr-60, Tyr-106, Ser-142	Gln-34, Hsd-58, Hsd-84, Arg-104, Tyr-136
4A1	Neu5Ac	−5.3	Gln-34, Gln-36, Ser-58, Hsd-60, Tyr-82, Tyr-84, Ala-112	Tyr-113, Gly-114
	Neu5Gc	−5.1	Gln-34, Tyr-82, Tyr-84	Gln-36, Ser-58, Hsd-60, Ala-112, Tyr-113, Gly-114
5G11	Neu5Ac	−5.6	Arg-61, Trp-82, Tyr-106, Gly-161	Hsd-108, Trp-130, Tyr-162, Tyr-163
	Neu5Gc	−5.9	Arg-61, Ser-84, Trp-130, Gly-161, Tyr-163	Glu-58, Asp-60, Trp-82, Tyr-106, Hsd-108, Tyr-162
6D2	Neu5Ac	−5.2	Asn-84, Tyr-106, Tyr-108, Val-136	Tyr-36, Tyr-58, Tyr-137, Gly-138
	Neu5Gc	−5.3	Tyr-32, Asn-34, Tyr-36, Tyr-106, Tyr-108	Tyr-58, Asn-84, Gly-138

## Data Availability

The data presented in this study can be made available upon request to the corresponding author. Data is not publicly available due to privacy reasons.

## Supplementary Materials

The following supporting information can be downloaded at: https://www.mdpi.com/article/10.3390/biotech13040046/s1, Figure S1: Putative ligand-binding sites of ccombody candidates (A) 2D8, (B) 4A1, (C) 5G11, and (D) 6D2 as predicted by the PrankWeb P2Rank server. Residues marked in red comprise the ccombody binding pocket. Figure S2. Competitive ELISA of ccombodies 2D8, 5G11, 6D2, and 4A1 using various monosaccharide haptens (A) Mannose, (B) *N*-acetylgalactosamine, (C) Glucose, (D) Galactose, and (E) *N*-acetylglucosamine.
