# Supplementary material for: Screening and Characterization of Sialic Acid-Binding Variable Lymphocyte Receptors from Hagfish
Source: BioTech (Basel). 2024 Nov 12;13(4):46. doi: 10.3390/biotech13040046 (PMC11586995; doi:10.3390/biotech13040046)
Supplement: Supplementary file 1 [file biotech-13-00046-s001.zip › biotech-3213083-supplementary.pdf]

BioTech

Supplementary Materials

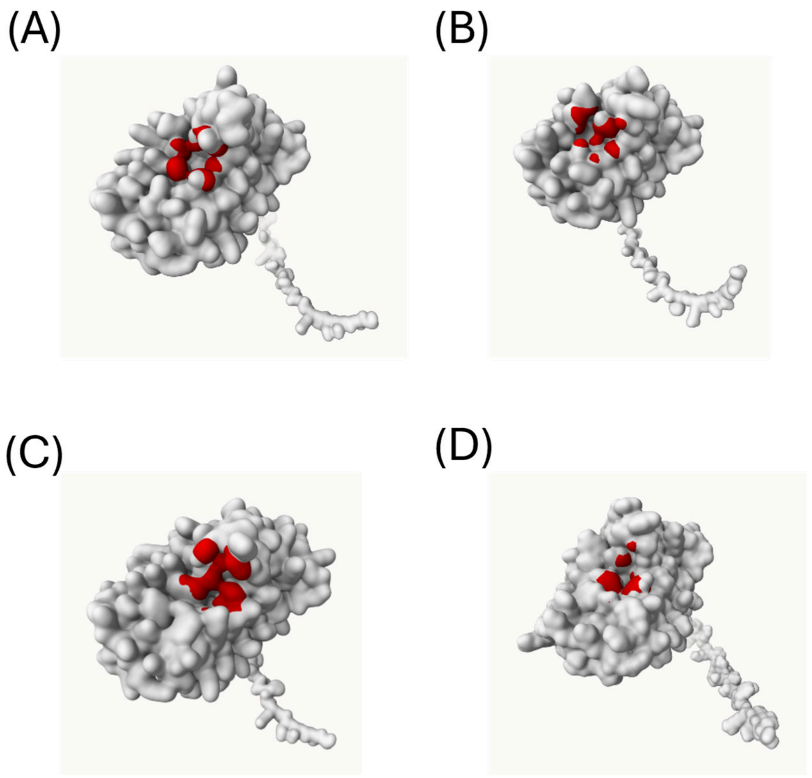

Figure S1. Putative ligand-binding sites of ccombody candidates (A) 2D8, (B) 4A1, (C) 5G11, and (D) 6D2 as predicted by the PrankWeb P2Rank server. Residues marked in red comprise the ccombody binding pocket.

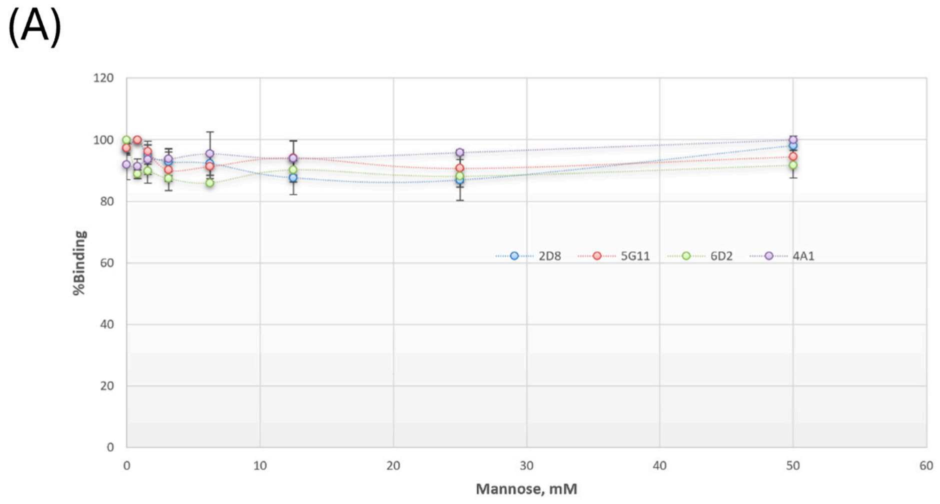

(B)

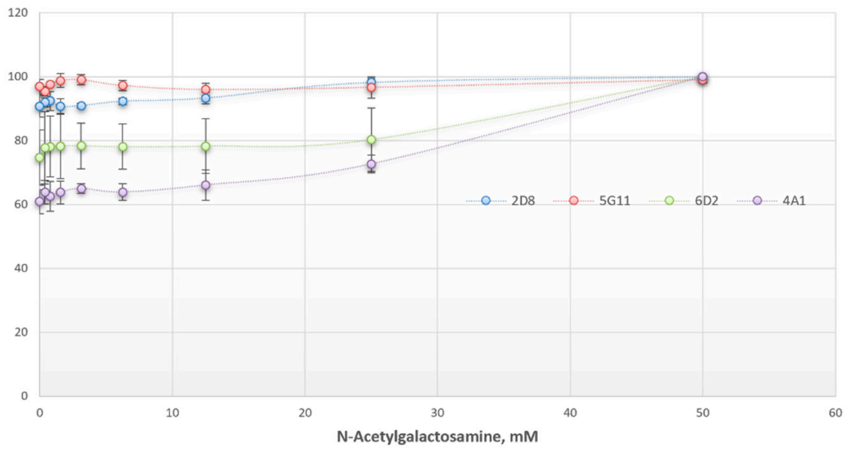
120
100
80
60
40
20
0
0
10
20
30
40
50
60
2D8
5G11
6D2
4A1
N-Acetylgalactosamine, mM

(C)

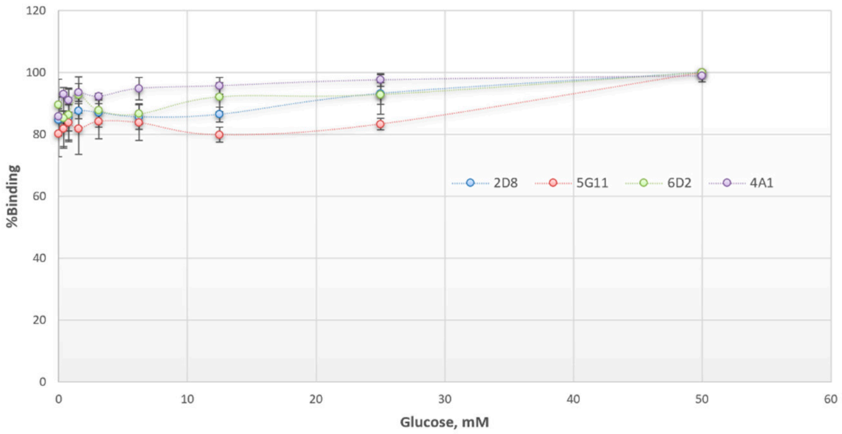
120
100
80
60
40
20
0
%Binding
0
10
20
30
40
50
60
2D8
5G11
6D2
4A1
Glucose, mM

(D)

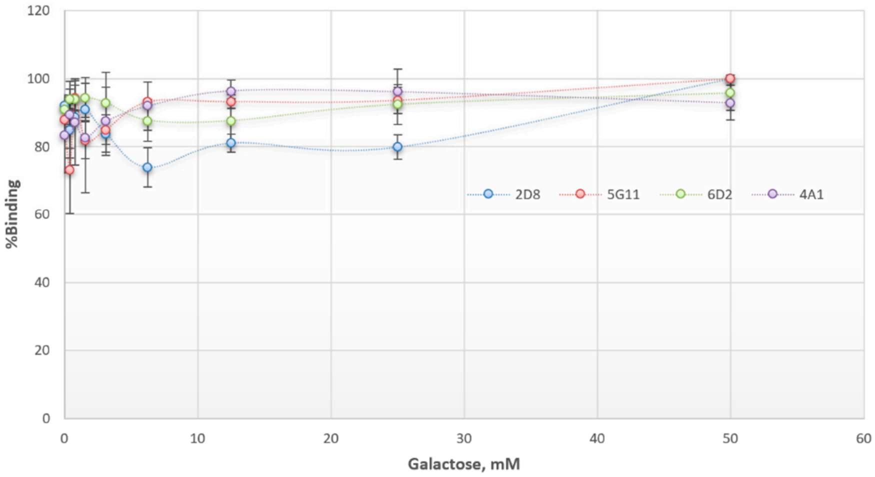
120
100
80
60
40
20
0
%Binding
0
10
20
30
40
50
60
2D8
5G11
6D2
4A1
Galactose, mM

(E)

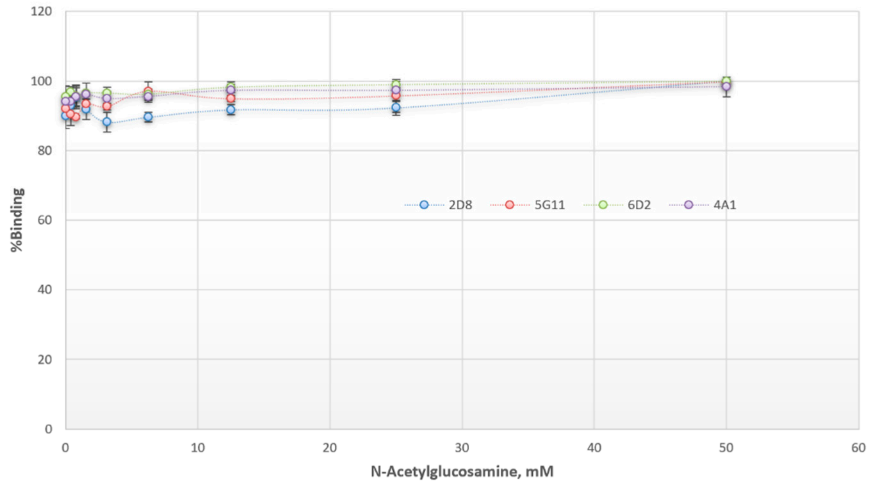

Figure S2. Competitive ELISA of ccombodies 2D8, 5G11, 6D2, and 4A1 using various monosaccharide haptens (A) Mannose, (B) *N*-acetylgalactosamine, (C) Glucose, (D) Galactose, and (E) *N*-acetylglucosamine.
